# A global urban tree leaf area index dataset for urban climate modeling

**DOI:** 10.1038/s41597-025-04729-y

**Published:** 2025-03-12

**Authors:** Wenzong Dong, Hua Yuan, Wanyi Lin, Zhuo Liu, Jiayi Xiang, Zhongwang Wei, Lu Li, Qingliang Li, Yongjiu Dai

**Affiliations:** 1 Southern Marine Science and Engineering Guangdong Laboratory (Zhuhai), Guangdong Province Key Laboratory for Climate Change and Natural Disaster Studies, School of Atmospheric Sciences, Sun Yat-sen University, Zhuhai, China; 2 College of Computer Science and Technology, Changchun Normal University, Changchun, China

**Keywords:** Climate and Earth system modelling, Projection and prediction

## Abstract

Urban trees are recognized for mitigating urban thermal stress, therefore incorporating their effects is crucial for urban climate research. However, due to the limitation of remote sensing, the LAI in urban areas is generally masked (e.g., MODIS), which in turn limits its application in Urban Canopy Models (UCMs). To address this gap, we developed a high-resolution (500 m) and long-time-series (2000–2022) urban tree LAI dataset derived through the Random Forest model trained with MODIS LAI data, with the help of meteorological variables and tree height datasets. The results show that our dataset has high accuracy when validated against site reference maps, with R of 0.85 and RMSE of 1.03 m^2^/m^2^. Compared to reprocessed MODIS LAI, our modeled LAI exhibits an RMSE ranging from 0.36 to 0.64 m^2^/m^2^ and an R ranging from 0.89 to 0.97 globally. This dataset provides a reasonable representation of urban tree LAI in terms of magnitude and seasonal changes, thereby potentially enhancing its applications in UCMs and urban climate studies.

## Background & Summary

Urban trees have been recognized for their ability to mitigate the urban thermal stress. Numerous field experiments and studies employing remote sensing techniques have evaluated and demonstrated that^1–4^. Their roles encompass effectively cooling urban surface by shading and evapotranspiration^5,6^ and influencing canyon wind and turbulent transport^7,8^. Additionally, it impacts anthropogenic energy use^9,10^ and can delay stormwater peak flow by intercepting precipitation^11–13^. Given the importance of urban vegetation, Urban Canopy Models (UCMs), as essential tools for urban climate research, have made substantial progress in modeling urban vegetation, particularly urban trees which have a more pronounced impact on local climate than shorter vegetation. Several models have successfully integrated trees into UCMs to explore their influence on mesoscale land–atmosphere exchange processes^14–16^, and in UCMs, the leaf area index (LAI) of the tree canopy is a critical structural attribute.

In recent years, advancements in remote sensing technology have significantly enhanced the monitoring of LAI in natural vegetation^17^. Since the 1980s, numerous satellite-based LAI datasets have been produced, including Global Inventory Modeling and Mapping Studies (GIMMS) LAI^18,19^, reprocessed Moderate Resolution Imaging Spectroradiometer (MODIS) LAI^20,21^, Global LAnd Surface Satellite (GLASS)^22,23^, GlOBMAP^24^, GEO^25,26^. These datasets, derived from AVHRR (Advanced Very High Resolution Radiometer) or MODIS, provide a wealth of valuable information and have been extensively used in General Circulation Models (GCMs) or Land Surface Models (LSMs).

**Fig. 1:**
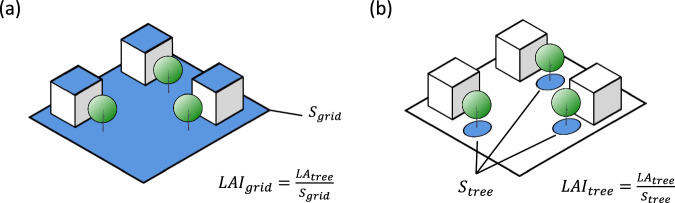
Different definitions of LAI: (**a**) grid LAI as used by satellite remote sensing (${LA}{I}_{{grid}}$) and (**b**) urban tree LAI as applied in UCMs (${LA}{I}_{{tree}}$). $L{A}_{{tree}}$, ${S}_{{grid}}$ and ${S}_{{tree}}$ represent the green tree leaf area, the grid area, and the total tree-covered area, respectively.

However, the limitations of passive optical detectors currently pose challenges in accurately estimating LAI in urban pixels, including building occlusion and the mixed pixels of artificial surfaces and vegetation, which affecting the reception and inversion of relevant band remote sensing signals. As a result, LAI values in urban areas are often masked or inadequately captured, such as MODIS LAI. Furthermore, the coarse resolution of AVHRR data (about 10 km) makes it unsuitable for capturing the intricate characteristics of urban vegetation dynamics and greening trend, due to urban constitute only a small fraction of the land area. GLASS v6 employs a machine learning method to generate global LAI data, including urban areas^22^. However, it’s important to note that this LAI represents “grid LAI”, i.e. the green leaf area per unit ground area (Fig. 1a), which can’t be directly applied in UCMs. According to its definition and the characteristics of remote sensing observations, this LAI represents the average distribution of leaf area evenly distributed across all leaves within the whole grid. Alternatively, UCMs generally require the “tree LAI”^27^, i.e. the green leaf area per unit tree-covered area (Fig. 1b), as UCMs need this to calculate the shading effects of trees on buildings or the ground. Consequently, due to issues related to the definition of LAI, the existing satellite-based datasets may not fully meet the requirements of UCMs for modeling urban trees.

While lidar technology is effective in obtaining accurate tree LAI data in urban areas^28,29^, it has obvious limitations in acquiring long-term and large-scale datasets. Currently, the ability to model the effects of urban trees on a global or regional scale is still constrained by the insufficient availability of urban tree LAI data, and urban tree LAI is commonly derived using preset values, knowledge-based assumptions, or empirical models^30–34^ for UCMs. There is a growing demand for more precise and detailed data that can effectively represent the phenological characteristics of urban vegetation to realistically resolve the influence of urban trees on urban radiation exchange and transpiration in UCMs. Therefore, to bridge the existing gap in urban tree LAI data and enhance the support for urban modelling, it is valuable to develop a specific LAI dataset tailored for urban tree modelling.

In this context, we aim to generate high-resolution urban tree LAI data to facilitate tree modeling in UCMs at various scales. We developed a machine learning models that utilize monthly minimum air temperature, vapor pressure, monthly incident shortwave radiation, monthly maximum air temperature, precipitation^35^, tree height^36^ and vapor pressure deficit as input features, with reprocessed MODIS LAI data^20^ as target for model training and validation. First, we employ the Random Forest (RF) model alongside the aforementioned datasets to model LAI in natural trees. Subsequently, we validate the modelled LAI against 14 ground LAI reference maps and reprocessed MODIS LAI data. The modeled LAI, at both site and global scales, demonstrated high accuracy. Compared to the reference maps, R and RMSE of modelled LAI are 0.85 and 1.03 m^2^/m^2^, respectively. When using reprocessed MODIS LAI as the benchmark, RMSE ranged from 0.36–0.64 m^2^/m^2^, and R ranged from 0.89–0.97 at the global scale. Based on the above results, we conclude that the model can accurately simulate LAI. Finally, the trained RF model is used to predict urban tree LAI, showing a reasonable representation of urban tree LAI in terms of both the magnitude and seasonal variations. This urban tree LAI dataset would enable more accurate and detailed representation of tree characteristics within urban areas, contributing to improved precision and reliability in urban tree modelling.

## Methods

In this study, we use an Automated Machine Learning library called FLAML^37^ as our machine learning model. FLAML is a lightweight Python library designed for efficient automation of machine learning and AI operations. It automates workflow based on machine learning models and optimizes their performance. We selected two machine learning model, RF^38^ and Light Gradient Boosting Machine (LightGBM)^39^ and chose the best one as the final model. Both RF and LightGBM are machine learning methods grounded in the ensemble learning concept and integrate multiple independent weak learners to enhance overall fitting capabilities. RF employs the bagging strategy, randomly selecting training samples and features during the construction of decision trees. Iteratively, it identifies the optimal split feature and split point from the selected dataset for each regression decision tree. The final prediction is based on the average of predictions from all regression decision trees. Zhang *et al*.^40^, successfully employed the RF model to predict LAI for four main plant functional types in Northern America. LightGBM is based on boosting algorithm and emphasizes efficiency and speed through innovative algorithms and optimizations. In model building, the parameters optimized for RF include *n_estimators*, *max_features*, and *max_leaves*, while the parameters optimized for LightGBM include *n_estimators*, *num_leaves*, *min_child_samples*, *learning_rate*, *log_max_bin*, *colsample_bytree*, *reg_alpha*, and *reg_lambda*. We did not specify parameter ranges, but instead set a maximum training time of 1800 seconds for the model to find the best hyperparameters.

Table 1 shows the explanatory variables used for the FLAML. Most variables were sourced from WorldClim v2.1, a dataset that provides spatially interpolated monthly climate data for global land areas at a high spatial resolution of 1 km. This includes monthly temperature (minimum, maximum, and average), precipitation, solar radiation, vapor pressure, and wind speed^35^. Furthermore, under identical environmental conditions, urban trees may exhibit different growth patterns compared to those in natural environments (e.g., urban trees are often shorter). To capture these differences and considering the documented relationship between LAI and tree height in previous studies^17,29,41^, tree height was included as an explanatory variable. For 1 km spatial resolutions, WorldClim v2.1 only provides historical climatology data, the model was trained for each month across different years using 1 km resolution climatology data, the primary difference between each model stems from varying target LAI which was used to represent annual variation. Although CRU-TS-4.06 provides downscaled data from 1969 to 2021, its resolution is limited to 2.5-minute, which is comparatively coarse. Consequently, the model constructed by CRU-TS-4.06 is inferior to that constructed with WorldClim v2.1 climatology data, additionally, since CRU-TS-4.06 only provides data on precipitation, maximum and minimum temperatures, and lacks data on radiation or vapor pressure, we still use WorldClim as the training data to maintain consistency. Moreover, the results indicate that WorldClim can reflect the temporal variation of LAI. Since WorldClim v2.1 does not provide vapor pressure deficit (VPD), we calculated it following Zhang’s method^40^, the equation is as follow:

1 $${VPD}=\frac{1}{2}\cdot 0.6108\cdot ({e}^{\frac{17.269\cdot {T}_{\max }}{237.3+{T}_{\max }}}+{e}^{\frac{17.269\cdot {T}_{\min }}{237.3+{T}_{\min }}})-{VP}$$

where *T*_*max*_ and *T*_*min*_ are monthly maximum and minimum air temperature (°C), respectively, VP is vapor pressure (Kpa). The tree height data were obtained from the global canopy height map developed by Lang *et al*.^36,42^. These data were derived from the Global Ecosystem Dynamics Investigation space-borne LiDAR mission, complemented by dense optical satellite images from Sentinel-2, achieving a resolution of 10 m. Additionally, MODIS Land Cover Type (MCD12Q1 V6.1)^43^ was employed to account for land cover changes. It is worth noting that the coverage of MCD12Q1 V6.1 extends from 2001 to 2022. Therefore, for the year 2000, we utilized the data from 2001, all details are shown in Table 1.

**Table 1:** Details of the explanatory variables for training the machine learning model.

Explanatory variables	Description	Temporal resolution	Spatial resolution	Reference
Htop	ETH tree height	2020	10 m	Lang *et al*.^36^
LAI	Reprocessed MODIS LAI	2000–2022	500 m	Lin *et al*.^20^
Tmax	Monthly maximum air temperature	1970–2000 climatology	1 km	Fick and Hijmans^35^
Tmin	Monthly minimum air temperature	1970–2000 climatology	1 km	
SW	Monthly incident shortwave radiation	1970–2000 climatology	1 km	
Prec	Monthly precipitation	1970–2000 climatology	1 km	
VP	Vapor pressure	1970–2000 climatology	1 km	
VPD	Vapor pressure deficit	1970–2000 climatology	1 km	Zhang *et al*.^40^

Given the scarcity of urban *in-situ* observational data for LAI, we chose satellite-based LAI as the target variable. In this study, the reprocessed MODIS LAI V6.1^20^ was utilized as the target variable for the RF and LightGBM model. The reprocessed MODIS LAI V6.1 covers the period from 2000 to 2022, with a temporal resolution of 8-day and a spatial resolution of 500 m. It was derived from the MCD15A2H V6.1^44^ using the modified temporal spatial filter method to fill the gap and processed the low-quality data. Compared to the original MODIS LAI, the reprocessed data exhibits better continuity in both the temporal and spatial domains. It also has been validated with *in-situ* LAI observations and demonstrates a good performance with various LAI reference maps.

Aiming to derive urban tree LAI, we carefully selected input feature and target data. First, we partitioned the global data into 5° × 5° region, excluding regions without land area, the global data was divided into 1447 regions. Subsequently, to better represent LAI in urban areas, we restricted our training data to natural trees surrounding urban regions, thereby excluding 5° × 5° regions without urban areas. This refinement ensured that only regions containing urban areas were retained, ultimately resulting in approximately 711 regions being selected as training areas. Finally, we simulated the LAI of natural tree in the 711 regions. Meteorological variables and tree height were employed as explanatory variables, with reprocessed MODIS LAI as the target. All samples were randomly divided into training (10%) and test (90%) groups. It is important to note that the samples were selected based on IGBP classifications 1–5, which represent evergreen needleleaf tree (NET), evergreen broadleaf tree (BET), deciduous needleleaf tree (NDT), deciduous broadleaf tree (BDT) and mixed forest (MF). Since our objective is to obtain the tree LAI in urban areas, the model was exclusively trained using tree LAI data. In addition, due to the training error of the model, and it was trained at a monthly scale, a simple moving average (SMA) method was used to buffer the fluctuations and prevent discontinuity of LAI values between adjacent months. The SMA equation is as follow:

2 $${LA}{I}_{n}^{{\prime} }={\rm{SMA}}\left({LA}{I}_{n},N\right)$$

Where ${LA}{I}_{n}$ is LAI of the month $n$ predicted by RF model, $N$ is the moving windows and we set it as 3, therefore, the final LAI of month $n$ (${LA}{I}_{n}^{{\prime} }$) is the average of ${LA}{I}_{n-1}$, ${LA}{I}_{n}$ and ${LA}{I}_{n+1}$. The model’s performance was evaluated at site (Table 2) and global scale using five error metrics: R^2^, R, RMSE, MBE and MAE. These evaluations aimed to determine whether the model could accurately extrapolate urban tree LAI. If the model demonstrated adequate performance, it was then utilized to predict urban tree LAI. LAI reference maps were collected from GBOV^45^, VALERI^46^, and Boston University^47,48^, all with IGBP land cover types 1–5, because we only train and predict LAI on the above types of grids. It should be noted that all sites provide 8-day LAI and may include missing values within a month. Since the predicted LAI is presented on a monthly basis, we utilized all available data to calculate the monthly LAI of the reference map for comparison with the predicted LAI. After the urban tree LAI prediction is completed, it is used to calculate urban tree Stem Area Index (SAI) using Zeng’s method^49^, the iterative equation for each month of one year is as follows:

3 $${SA}{I}^{n+1}=\max \left({SA}{I}^{n}\cdot {r}_{{tn}}+0.5{LA}{I}_{{diff}},{SA}{I}^{n}\right)$$

Where ${r}_{{\rm{tn}}}$ is the residual SAI retention (0.5), ${{LAI}}_{{\rm{diff}}}$ represents the LAI difference of two adjacent months, the superscript $n$ denotes iteration step of SAI, and the initial value (${SA}{I}^{0}$) is:

4 $${SA}{I}^{0}={SA}{I}^{{\prime} }\frac{{LA}{I}_{\max }}{{LA}{I}_{\max }^{{\prime} }}$$

**Fig. 2:**
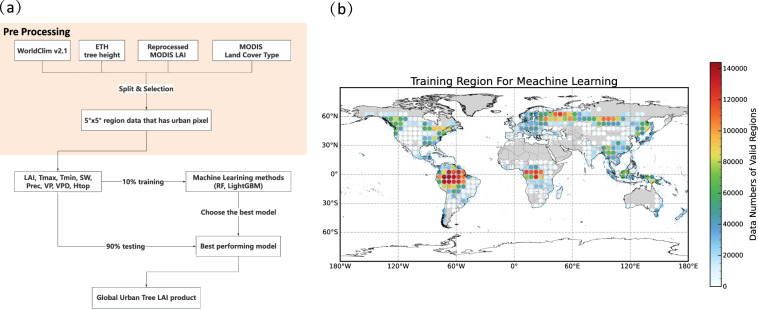
(**a**) Workflow of data preprocessing, machine learning model construction, and (**b**) the sample size for training in each 5° × 5° region.

Where ${LA}{I}_{\max }$ is the maximum LAI value in a year, ${SA}{I}^{{\prime} }$ and ${LA}{I}_{\max }^{{\prime} }$ are both constants, with values 1 and 5.5 m^2^/m^2^ respectively. The stopping criterion for the iterative calculation is met when the sum of the differences in SAI values between two iterations across 12 months is less than 1.0E-6. The methodology, including its key steps, is shown in Fig. 2.

**Table 2:** Characteristics of the 14 validation sites (Land Cover Type is derived from MCD12Q1).

Site	Land Cover Type	Lat	Lon	Source
BART	BDT	44.0639	−71.2873	GBOV^45^
Counami	BDT	5.34	−53.24	VALERI^46^
HAIN	BDT	51.079	10.452	GBOV^45^
HARV	BDT	42.5378	−72.1715	GBOV^45^
Larose	BDT	45.38	−75.22	VALERI^46^
Nezer	NET	44.57	−1.04	VALERI^46^
ORNL	BDT	35.96412	−84.2826	GBOV^45^
SCBI	BDT	38.89292	−78.1395	GBOV^45^
STEI	BDT	45.50894	−89.5864	GBOV^45^
TALL	BDT	32.95046	−87.3933	GBOV^45^
TUMB	NET	−35.65652	148.15163	GBOV^45^
UNDE	BDT	46.23396	−89.5375	GBOV^45^
Ruokolahti	NET	61.53	28.71	Boston University^47,48^
Puechabon	NET	43.72	3.65	VALERI^46^

## Data Records

The dataset is available at Zenodo (10.5281/zenodo.14709655)^50^. The global LAI data are stored in NetCDF files. Each file contains the monthly LAI (also SAI) for all months in a year, with the data dimension defined as “mon × lat × lon”. Different resolutions of data are provided in separate directories, named global_XX, in which all the filenames follow the convention of “Global_UrbanTree_LAI_XX_YYYY.nc”, where XX represent the resolution of the data and YYYY indicates the year.

## Technical Validation

**Fig. 3:**
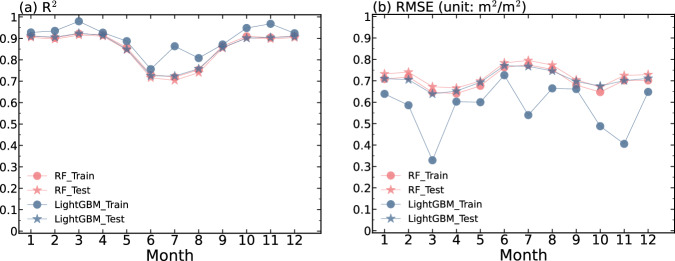
Performance of RF and LightGBM models in training and testing sets: (**a**) ${R}^{2}$, (**b**) RMSE.

First, we evaluated the performance of the two machine learning models to determine the most accurate algorithm for predicting LAI. Figure 3a demonstrates the performance of LightGBM and RF on training and test dataset of each month. The result shows that LightGBM exhibits a higher ${R}^{2}$ in training data than RF, however there are significant discrepancies between the test and training sets in certain months, and the ${R}^{2}$ in training data is close to RF. In contrast, for RF, the values for the test and training sets remain relatively consistent throughout. For RMSE, the results are similar to R. We also compare the annual predicted grid tree LAI in global of 2020. Generally, both models have a similar R (0.97) and MBE (around 0.1 ${m}^{2}/{m}^{2}$), but LightGBM has lower RMSE (0.45 ${m}^{2}/{m}^{2}$) than RF (0.51 ${m}^{2}/{m}^{2}$). However, LightGBM may has an overfitting problem, except that there are obvious differences between the training set and the test set as shown in Fig. 3b, LightGBM occasionally predicts negative LAI values or values outside the training set range (0–7 ${m}^{2}/{m}^{2}$) for some 500 m grids. Therefore, the RF model was deemed more stable and was consequently applied for LAI prediction.

**Fig. 4:**
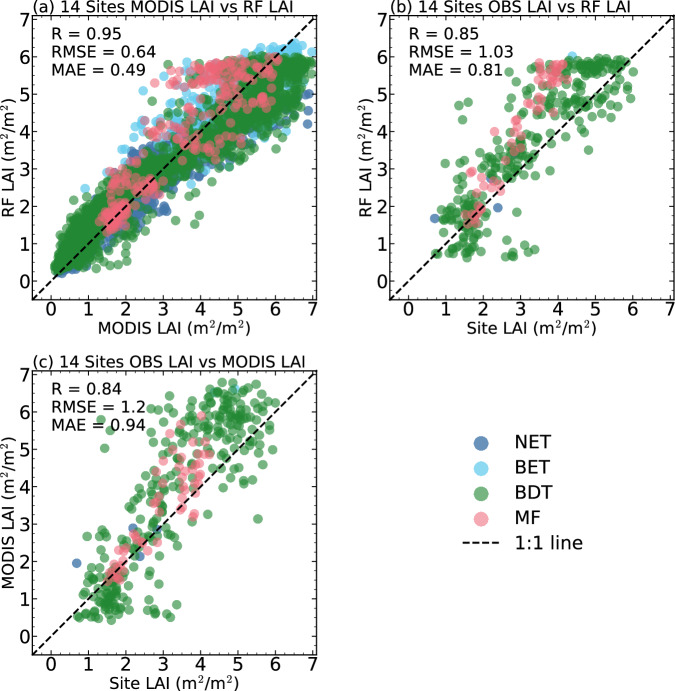
The scatter plot of (**a**) RF LAI and MODIS LAI, (**b**) RF LAI, (**c**) MODIS LAI and field LAI measurements for 14 sites. Sites with different land cover types are marked by colors, and the black dashed 1: 1 line is drawn.

After that, we evaluate the RF predicted LAI with MODIS V6.1 and all site observation LAI (Table 2). Figure 4a shows the validation result for the RF predicted and MODIS V6.1 LAI, indicating that RF predicted LAI exhibits a high accuracy, with an R of 0.95, an RMSE of 0.64 ${m}^{2}/{m}^{2}$, and a MAE of 0.49 ${m}^{2}/{m}^{2}$. Moreover, when assessed against field LAI measurements, the LAI produced by RF models also demonstrates high accuracy. The validation of the RF LAI revealed that the R reached 0.85, and the RMSE was approximately 1.03 ${m}^{2}/{m}^{2}$. This result closely mirrors the validation results between MODIS V6.1 LAI and the reference map LAI (Fig. 4c), suggesting that the errors between the model and the site observations mainly come from the training data.

**Fig. 5:**
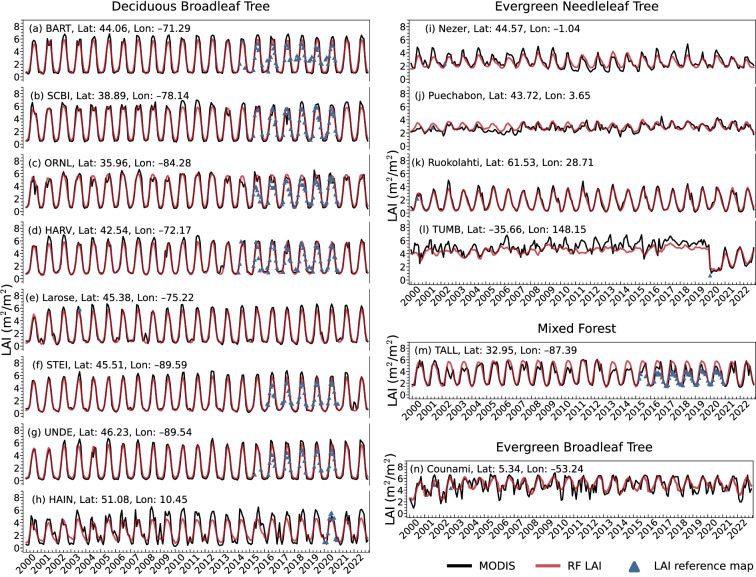
Temporal variations of the monthly RF, MODIS LAI compared with field measurements LAI for 14 sites during 2000–2022.

Figure 5 shows the validation for the temporal variations of the RF predicted LAI, MODIS V6.1 LAI and site observation LAI during 2000–2022. The time-series result shows that the RF predicted LAI is consistent with MODIS V6.1 LAI and accurately captures both the seasonal cycles and annual changes. For the BDT cover, RF predicted LAI are quite similar with site observation LAI and the RF predicted LAI are smoother, for example, at SCBI (Fig. 5b) and HARV (Fig. 5e), RF predicted LAI usually fluctuates less or not at all. For NET, BET and MF cover, although the reprocessed MODIS V6.1 LAI mitigated LAI mutations through the reprocessing algorithm, LAI may still fluctuate greatly between adjacent months. While the RF LAI demonstrates a smoother pattern, especially for BET. The LAI reference map coincides well with both the RF predicted and MODIS V6.1 LAI values at the BDT cover site, but at MF cover, the RF predicted LAI and MODIS V6.1 LAI are generally larger than observed values at summer. Moreover, Fig. 5l illustrates that it is indeed feasible to employ climate explanatory variables and different target LAI to train the model, thereby enabling it to capture interannual changes in LAI. Between 2019 and 2020, MODIS LAI at TUMB experienced a sudden decrease, and RF LAI also exhibited a similar change.

**Fig. 6:**
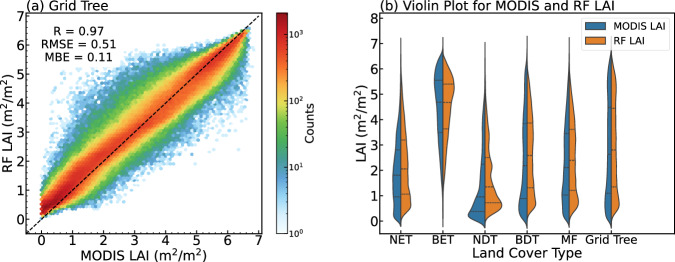
Direct validation of RF and MODIS LAI at a global scale: (**a**) a scatter plot comparison of the monthly RF and MODIS LAI for 2020, and (**b**) a violin plot illustrating the distribution of monthly LAI for each land cover type (both comparisons are at a 0.5° resolution).

The scatter plot of 2020 monthly grid tree LAI of RF predicted and MODIS V6.1 is shown in Fig. 6a, RF predicted LAI also shows a great performance in global scale. The result indicates that there is a good agreement between RF predicted LAI and MODIS LAI, with R of 0.97, RMSE of 0.51 ${m}^{2}/{m}^{2}$, and MAE of 0.11 ${m}^{2}/{m}^{2}$. We also conducted an evaluation of the predicted LAI for each land cover type, as shown in Fig. 6b. The LAI of each land cover type was slightly overestimated at low values. However, the distribution of predicted LAI is consistent with that of MODIS. It should be noted that we trained a single model to predict LAI for all land cover types, which could potentially result in biased predictions for specific types, such as NDT. However, our focus is on the urban tree LAI, from the results, the difference between the grid tree LAI predicted by RF and that from MODIS is within an acceptable range.

**Fig. 7:**
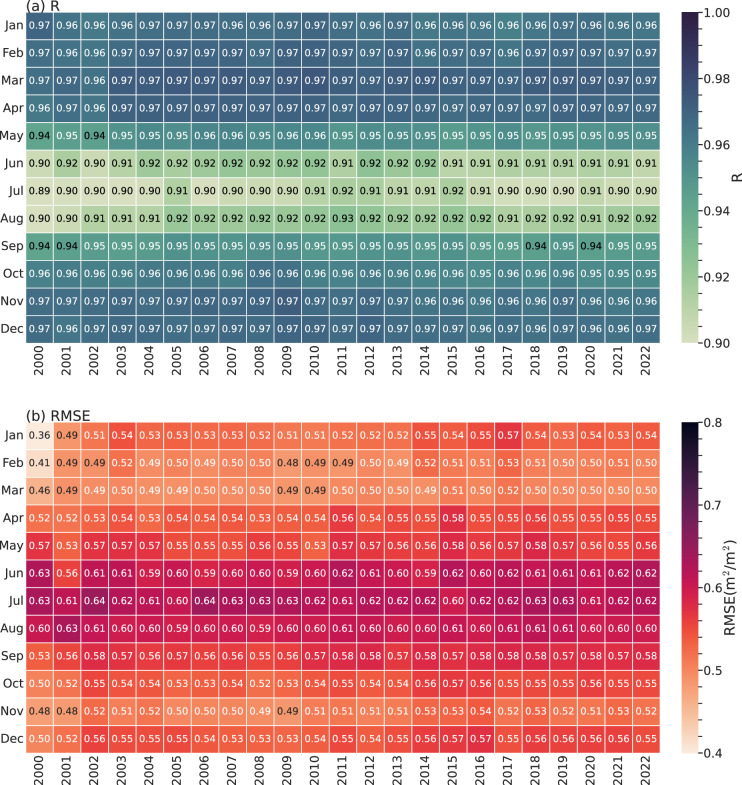
Global-scale validation of monthly RF and MODIS LAI during 2000–2022 using (**a**) R and (**b**) RMSE (comparisons conducted at a 0.5° resolution).

The heatmap of R and RMSE between monthly RF predicted and MODIS grid LAI from 2000 to 2022 are shown in Fig. 7. The result shows that the R of these two LAI range from 0.89–0.97 and RMSE range from 0.36–0.64 ${m}^{2}/{m}^{2}$. Although the model was trained using all the selected regions, the significant regional variations in the Northern Hemisphere during summer may have hindered the model from fully capturing all relevant features. Consequently, this season shows the highest RMSE and the lowest R values. However, the result demonstrates that the RF model can capture spatial and temporal variations in LAI well, it also proved that it is feasible to use target LAI values to represent annual variation even with limited training data. Based on the above results, we concluded that the RF model can reproduce the spatiotemporal variation in LAI data accurately in a masked urban grid, thereby fulfilling the requirements for model simulation.

Due to the lack of urban tree LAI observations, we assess the rationality of RF LAI data at urban sites sourced from Urban-Plumber2^51^. As shown in Table 3, our dataset demonstrates a reasonable magnitude and seasonal variation at these urban sites. In mid-to-high latitude urban sites dominated by deciduous broad-leaved and evergreen needle-leaved trees, the LAI generally peaks in summer, up to 4.9 ${m}^{2}/{m}^{2}$ at US-Minneapolis. For evergreen broad-leaved trees, e.g., AU-Preston, no significant seasonal changes were found. However, GLASS LAI generally has a lower value and no significant seasonal changes at these sites, mainly due to the high proportion of impervious and the low vegetation cover affecting the reception and inversion of relevant band remote sensing signals. For example, at US-Minneapolis, with 0.38 tree and 0.21 impervious cover exhibit a maximum LAI of approximately 3 ${m}^{2}/{m}^{2}$. In contrast, at other locations, the maximum LAI does not exceed 1 ${m}^{2}/{m}^{2}$, which is inconsistent with actual photography. In addition, while urban tree LAI exhibits similar growth patterns to natural tree LAI, differences in magnitude exist, with the LAI of natural trees typically being slightly higher than that of urban trees.

**Table 3:** Temporal comparison of the monthly RF, GLASS LAI of 9 urban sites during 2020, accompanied by corresponding images of each site, RF and GLASS LAI are the LAI of the pixel (500 m grid) where the flux tower is located and natural LAI is the weighted average of all-natural tree LAI within the 0.5° grid where the flux tower is located.

Site name	Photographs and monthly LAI		Properties	
	Eye level	LAI		
**1. AU-Preston**			Tree Type	BET
			%Tree	22.5
			%Impervious	62
**2. CA-Sunset**			Tree Type	NET
			%Tree	12
			%Impervious	68
**3. PL-Lipowa**			Tree Type	NET
			%Tree	16
			%Impervious	76
**4. SG-TelokKurau**			Tree Type	BET
			%Tree	11
			%Impervious	85
**5. UK-Swindon**			Tree Type	NET
			%Tree	9
			%Impervious	49
**6. US-Minneapolis**			Tree Type	BDT
			%Tree	38
			%Impervious	21

**Fig. 8:**
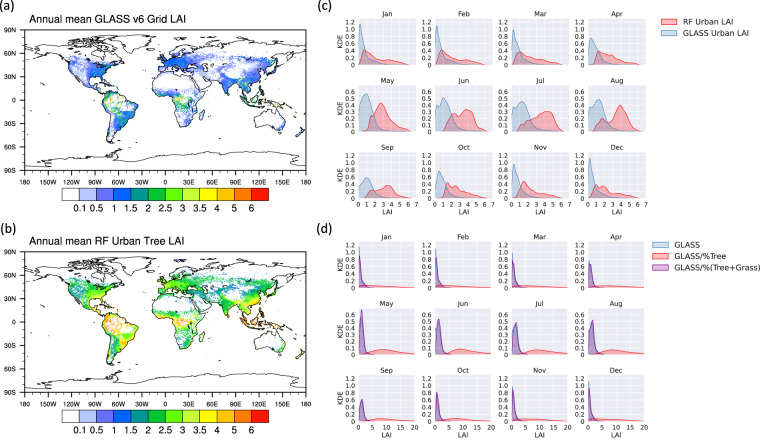
Global distribution of annual mean urban LAI of (**a**) GLASS and (**b**) RF, and the KDE distribution of monthly urban LAI of (**c**) RF and GLASS, and (**d**) the KDE distribution of original and adjusted (divided by the vegetation cover percentage) GLASS urban LAI (All shows are for 2020).

Figure 8 shows the LAI of predicted tree LAI and GLASS grid LAI and their LAI value distribution, respectively. As shown in Fig. 8a,b, RF predicted LAI generally exhibits a higher LAI value in urban areas due to it is specifically calculated for tree-covered areas. In contrast, satellite remote sensing in mixed pixels like urban areas may be influenced by building occlusion and get a lower value. As demonstrates by Fig. 8c, GLASS LAI typically ranges from 1–2 *m*^2^/*m*^2^ on a global scale, but RF predicted LAI shows a distinct seasonal variation, with LAI values typically ranging from 3 to 5 *m*^2^/*m*^2^ in summer and 1 to 3 *m*^2^/*m*^2^ in winter. And calculating LAI in vegetation-covered areas using vegetation coverage (tree and grass coverage) proves challenging (Fig. 8d), i.e. using LAI divided by the vegetation cover percentage. There was no significant change when the percentage of grass and trees was used, and an unrealistic LAI (>10 *m*^2^/*m*^2^) was obtained when only the trees cover is used.

## Usage Notes

A correct lower boundary condition is crucial for weather climate model. As LSMs have enhanced their spatial resolution to 9 km globally (e.g., ERA5-Land) or 1-2 km for specific regions (e.g., WRF), employing an appropriate urban parameterization scheme to differentiate between urban and rural areas and better representation of the urban environment is necessary. However, these require accurate global ancillary data, including building morphology and vegetation data in urban areas. Considerable effort has applied to produce global-scale building morphology data^52–54^. As demonstrate by Zhao *et al*.^55^, substantial urban afforestation has occurred across numerous cities, playing an important role in mitigating urban heat. In addition, previous studies have proved that integrating urban vegetation in UCMs is important for improving model performances^56–59^. Therefore, it is necessary to represent the influence of trees in the UCMs.

In this study, we used machine learning and remote sensing LAI data to generate an urban tree LAI dataset at 500 m from 2000 to 2022 for UCMs. Over the last decade, model community has made a great effort to improve and develop urban tree radiative model^7,14,16,34,60^. However, there remains a scarcity of LAI data suitable for meso and large-scale simulations in UCMs, some studies can only use preset values^27,32^. The 500 m urban tree LAI dataset generated in this study mainly focus on providing an essential dataset for urban land surface model, enabling them to simulate the influence of trees in urban environments across various temporal and spatial scales, particularly the shading effect. Additionally, this dataset can also be used to evaluate the urban greening, and its impact on urban climate^55^.

This dataset is highly recommended for users who wish to model the tree in UCMs, especially at a region-global with long time scale. When using this dataset, users need to make it clear that the definition of LAI for the data is the LAI only in tree-covered areas within urban. In other words, the urban tree LAI value represents green leaf area per unit tree-covered surface area within a 500 m grid, excluding buildings and other impervious surfaces. However, users can also calculate the 500 m grid LAI using tree cover percentage data, depending on their specific requirements.

In addition, this dataset, generated using machine learning methods, should be aware of the limitations of machine learning, it can’t always accurately model the variation in LAI, particularly for extreme values or so-called “tipping points“^61^. Secondly, we use natural tree LAI surrounding urban to extrapolate the urban LAI, while we incorporated tree height to account for differences between urban and natural trees and restricted the training area to resemble urban environments as closely as possible, data limitations, such as the availability of tree height data for only a single year, may introduce uncertainty into our model. Finally, as a reprocessing dataset, MODIS LAI, which serves as a target variable, also introduces additional uncertainty into the machine learning process. However, this dataset is produced for urban climate modeling at a medium-large scale, based on the accuracy assessment, it can be considered as input data for urban climate modeling.

All monthly 500 m, 0.05° and 0.5° resolution LAI data are stored in NetCDF files and can be accessed at 10.5281/zenodo.14709655^50^. Additionally, users can request coarse-resolution data at their desired spatial resolution and temporal coverage directly from the corresponding author. It is worth noting that the dataset will be updated as new or improved input data become available.

## Data Availability

The codes used in this study are available at https://github.com/tungwz/RF_LAI.
